# A comparison of chronic pain with and without neuropathic characteristics in a Hong Kong Chinese population: An analysis of pain related outcomes and patient help seeking behaviour

**DOI:** 10.1371/journal.pone.0204054

**Published:** 2018-10-24

**Authors:** Stanley Sau Ching Wong, Siu Wai Choi, Chi Wai Cheung

**Affiliations:** 1 Laboratory and Clinical Research Institute for Pain, Department of Anaesthesiology, The University of Hong Kong, Hong Kong, HKSAR; 2 Department of Oro-maxillofacial surgery, The University of Hong Kong, HKSAR; University of Tokyo, JAPAN

## Abstract

**Objective:**

In Western countries, chronic pain patients with neuropathic characteristics have more intense pain, greater negative impact in quality of life and worse psychological well-being. The aim of this study was to compare the outcomes, impact, and health seeking behaviours in Chinese chronic pain patients with and without neuropathic characteristics in Hong Kong.

**Methods:**

Random telephone survey was conducted on the general Hong Kong population, and based on the Nuprin Pain Report. Specific questions on chronic and neuropathic pain were included. Respondents with pain lasting three months or more were asked to indicate their two most painful sites. Chi-square and Mann-Whitney U-test were used to investigate differences between variables in patients with and without neuropathic characteristics. P<0.05 was regarded as significant.

**Results:**

The response rate was 32.3%. Chronic pain patients with neuropathic characteristics reported higher pain scores and longer duration of pain (p = 0.0001). They reported greater negative impact on work and effect on daily life (p = 0.0131); were significantly more likely to consult pain specialists (p = 0.0006), Chinese medicine practitioners (p = 0.0203), and psychiatrists (p = 0.0212); and were significantly less likely to be prescribed oral analgesics (p = 0.0226), to feel ‘very satisfied’ (p = 0.0263) with prescribed treatment and to find oral analgesics ‘very useful’ (p = 0.0215). There was no difference in oral analgesic medications taken.

**Conclusion:**

Chinese individuals having chronic pain with neuropathic characteristics had worse pain related outcomes. Differences in help-seeking behaviour were observed. Lack of appropriate analgesic prescription suggests that identification and management of chronic neuropathic pain in Hong Kong needs to be improved.

## Introduction

Neuropathic pain is defined as pain caused by a lesion or disease of the somatosensory system [1]. It has an estimated prevalence of between 6.9–10% [2]. Neuropathic pain is an important public health problem. It has a negative impact on quality of life, function, sleep, and depression [3]. Chronic neuropathic pain is also associated with increased health care utilization and increased drug prescription [3, 4]. Neuropathic pain is often chronic and difficult to treat, and has a greater negative impact than non-neuropathic pain [1, 5]. The mechanism of pain underlying neuropathic pain is different from non-neuropathic pain [6]. Therefore, it should be managed as a distinct clinical condition.

Previous studies have compared chronic pain with neuropathic characteristics (NP) with chronic non-neuropathic pain (non-NP). Individuals with chronic neuropathic pain were associated with more severe pain, longer duration of pain, pain in more body locations, lower quality of life, worse sleep quality, increased symptoms of anxiety and depression, and greater use of health care resources [3, 4, 7–9]. In a cross sectional telephone interview, we reported that the prevalence of pain with neuropathic characteristics (acute and chronic) was 9.03% in a Chinese population in Hong Kong [10]. In addition, 14.7% of Chinese patients suffering from chronic pain in Hong Kong had neuropathic characteristics [10]. These figures indicate that this is a significant health care problem in Chinese people. Currently, there is no information comparing the severity, outcomes, impact, and help seeking behaviours between individuals with chronic NP and chronic non-NP in a Chinese population. Population studies on chronic pain with neuropathic characteristics in a Chinese population were previously difficult to conduct due to the absence of validated neuropathic pain questionnaires in the Chinese language. Currently, the Identification Pain Questionnaire for neuropathic pain has been validated in Chinese, and can be used to identify individuals suffering from pain with a significant neuropathic component [11]. The aim of this cross sectional study was to compare the outcomes, impact, and help seeking behaviours between individuals with chronic NP and chronic non-NP in a Chinese population in Hong Kong.

## Materials and methods

This cross-sectional survey conducted on the general population of Hong Kong was approved by the Institutional Review Board of the University of Hong Kong/Hospital Authority Hong Kong West Cluster and registered at ClinicalTrials.gov (registration number NCT01878877). The study was conducted via a telephone survey adapted from the Nuprin Pain Report, especially developed for this current investigation, and tailored to the Cantonese speaking population, based on a similar study conducted by our department in 1999 [12, 13]. The full surveys (in English and Chinese, S1 Appendix and S2 Appendix) included specific questions to assess the prevalence of chronic pain with neuropathic characteristics (NP) as well as pain-relieving modalities. Changes made to the pain categories included in the Nuprin Pain Report were the inclusion of oro-facial pain, other pain sites not listed, and difficult to describe pain sites. Qualitative aspects on perceived causes of pain, treatment sought and satisfaction with treatment were included in this current survey. According to the 2012 Hong Kong Census, Cantonese was the most common language used in Hong Kong, therefore, only those who could understand and speak Cantonese were included in this survey [14]. Randomization was achieved by using telephone numbers generated using a computer program. Telephone calls were made in the evening on weekdays, between the hours of 18:00 to 22:30, and if more than one adult member of the household was available for interview, the adult with the nearest upcoming birthday was selected for interview.

All the interviews, which required approximately 15 minutes to complete, were performed by trained interviewers from the Public Opinion Program (POP) at the Faculty of Social Sciences (The University of Hong Kong), from May 30^th^ 2013 until June 28^th^ 2013. Verbal consent was obtained before the interview.

Respondents who indicated that they had suffered chronic or recurrent pain for a period of at least three months met the study definition of chronic pain. Respondents who met the chronic pain criteria were asked to name the two most painful sites, and to quantify their pain using the numerical rating scale, (NRS), with a score of 0 meaning no pain, and 10 being the worst pain imaginable. The two most painful sites were considered for further analysis regardless of how many pain sites the respondents actually reported.

The Identification Pain Questionnaire for neuropathic pain has been translated and validated in Chinese and this Chinese version was used to identify respondents who suffered from pain with neuropathic characteristics [11]. The questions were: 1) Did the pain feel like pins and needles 2) Did the pain feel hot/burning 3) Did the pain feel numb 4) Did the pain feel as though you had electric shocks 5) Is the pain made worse with the touch of clothing or bed sheets 6) Is the pain limited to your joints? Respondents would score one mark if they answered ‘yes’ to questions 1 to 5, and would have one mark deducted from their score if they answered ‘yes’ to question 6. A score of three or more indicated the presence of pain with neuropathic characteristics (NP) [11]. This questionnaire was chosen for its ease of use over the telephone.

Sociodemographic data collected included gender, self-reported age, marital status, type of employment, employment status (whether in current employment) and educational level. The type of medical treatment sought, satisfaction with treatment, self-treatment and type of medications used were also recorded.

All analyses were conducted using SPSS (version 21.0.0). Demographic data from respondents who indicated that they had suffered from recurrent or chronic pain for at least three months, and those who were identified as suffering from neuropathic pain, were described using means, standard deviations and percentages, as appropriate. Differences in demographic data between those who were suffering from chronic, non-neuropathic pain and those who were suffering from chronic neuropathic pain were investigated using chi-squared test, or Mann-Whitney U test, as appropriate. Binary logistic regression was then used to investigate which demographic characteristics could predict chronic neuropathic pain. Chi-square test was used to investigate differences between high pain scores, defined as ≥ 7 on the NRS, reported pain sites, the most painful sites, treatment sought both from professionals and self-treatment, medications taken, satisfaction with treatment, self-perceived cause of pain, effect of pain on work, and effect of pain on daily life and sick leave taken between those with and without neuropathic pain. Mann-Whitney U test was used to investigate differences in continuous outcomes, including highest and average pain score and duration of pain suffering between those with and without neuropathic pain characteristics. A p value of 0.05 was regarded as significant.

As was reported in our previous publication, the target sample size was calculated on the presumption of a 20% prevalence rate in chronic pain [10]. The 20% chronic pain prevalence was estimated as having doubled from the 10% prevalence of chronic pain estimated from a population based study collected in 1999 [13]. Based on a 95% confidence level with ±2.5% margin of error, it was calculated that a minimum of 1000 successful interviews was required.

## Results

### Response rate and characteristics of respondents

A total of 4858 successful calls were made and 1570 respondents interviewed, corresponding to a response rate of 32.32%. This was lower than the response rate of 47.7% in the 1999 survey, but the current study still reaches the required power. Out of the 1570 respondents interviewed in 2013, 715 (45.54%) were male and 855 (54.46%) were female.

A total of 468 respondents were identified to have chronic pain. Out of these respondents with chronic pain, 80 were identified as having pain with neuropathic characteristics (NP). The demographic data of those with and without neuropathic pain characteristics are shown in Table 1. There were a significantly larger proportion of females with chronic NP, compared to those with chronic non-NP (80.0% vs 70.0%, p = 0.003). There were no other demographic differences between the two groups, with the exception that a higher proportion of respondents with chronic NP reported being unemployed due to health problems (11.3% vs 1.3%, p = 0.0001). A significantly higher proportion of respondents suffering from chronic NP reported feeling under ‘much more pressure than others’ (20.3% vs 7.4%, p = 0.0012) (Table 1).

**Table 1:** Socio-demographic information of respondents with chronic pain with neuropathic characteristics (NP) and chronic non-neuropathic pain (non-NP).

	Chronic NP (n = 80)	Chronic non-NP (n = 388)	p value
**Sex**			
Male	20	30	0.1257
Female	80	70	0.3361
**Age** [mean (SD)]	53.56 (15.4)	55.07 (14.39)	0.434
**Marital status (%)**			
Single	15	16	1
Married	72.5	71.4	0.8924
Divorced	5	5.2	1
Widowed	7.5	6.2	0.6201
Not known	0	1.3	-
**Educational level (%)**			
Primary school or below	30	28.08	0.7856
Secondary school	53.75	48.2	0.3915
University or above	15	23.2	0.1362
Unknown	1.25	0.52	0.4309
**Occupational status (%)**	** **	** **	** **
Full time	21.3	39.7	0.0021
Part time	13.8	10.1	0.3236
Unemployed	-	1.8	-
Homemaker	18.8	17.5	0.7506
Student	3.8	1.3	0.1413
Part time student	-	1.2	-
Retired	31.0	27.1	0.4932
Unemployed due to health problems	11.3	1.3	0.0001
**Occupational type (%)**	** **	** **	** **
Professional	7.5	16.1	0.0549
Administrative and service industry	20	23.64	0.5603
Manual labour work	7.5	8.57	1
Student	3.75	1.82	0.3868
Homemaker	18.75	17.66	0.8725
Others	42.5	32.21	0.0915
**Perceived Pressure (%)**			
I’m under much more pressure than others	20.3	7.4	0.0012
I’m under more pressure than others	27.8	21.9	0.3026
I’m under the same pressure as others	32.9	43	0.1038
I’m under less pressure than others	19	27.7	0.1227

Values in %.

### Risk factors for chronic neuropathic pain

Binary logistic regression identified female sex (odds ratio (OR), (95% C.I), 3.714 (1.585 to 8.707) p = 0.003), working part time (OR (95% C.I), 2.503 (1.034 to 6.061) p = 0.042), being a full-time student (OR (95% C.I), 8.211 (1.300 to 51.852) p = 0.025), and feeling very much under pressure (OR (95% C.I), 4.000 (1.764 to 9.069) p = 0.001) as risk factors for having chronic NP (Table 2).

**Table 2:** Socio-demographic correlation with chronic pain with neuropathic characteristics.

Characteristic	Odds ratio (95% CI)	P value
***Age***		
18–29	reference	
30–49	1.719 (0.290 to 10.203)	0.551
Aged 50 or above	2.432 (0.399 to 14.813)	0.335
***Gender***		
Male	reference	
Female	3.714 (1.585 to 8.707)	0.003
***Marital status***		
Single	reference	
Married/cohabit	1.042 (0.404 to 2.689)	0.932
Divorced/separated	0.350 (0.069 to 1.783)	0.206
Widowed	1.028 (0.279 to 3.790)	0.967
***Education level***		
None	reference	
Completed primary education	1.119 (0.334 to 3.744)	0.856
Completed middle school	1.358 (0.397 to 4.647)	0.626
Completed high school	1.414 (0.411 to 4.865)	0.583
Matriculation	0.455 (0.065 to 3.212)	0.43
Undergraduate	2.205 (0.460 to 10.569)	0.323
Postgraduate	0.559 (0.122 to 2.560)	0.454
***Occupation status***		
Working full time	reference	
Working part time	2.503 (1.034 to 6.061)	0.042
Unemployed	1.498 (-)	1
Home-maker	2.396 (0.692 to 8.298)	0.168
Student (full time)	8.211 (1.300 to 51.852)	0.025
***Occupation***	*** ***	
Professional	reference	
Clerical and service industry	1.339 (0.406 to 4.416)	0.632
Manual	1.618 (0.387 to 6.769)	0.51
Others	0	1
***Perceived pressure***		
Feel under less pressure than peers	reference	
Feel somewhat under pressure	1.117 (0.565 to 2.207)	0.751
Feel the same pressure as peers	1.855 (0.906 to 3.799)	0.091
Feel very much under pressure	4.000 (1.764 to 9.069)	0.001

### Location and number of severe pain sites

Average pain scores were similar between chronic non-NP and chronic NP respondents. Highest pain score was significantly higher in respondents with chronic NP (7.75 (1.698) [3–10] versus 6.43 (2.141) [1–10] (p = 0.0001) (Table 3). Respondents with chronic NP were more likely to report having pain sites with a pain score of ≥7 (overall p = 0.0001) (Table 3). Average pain scores across different body locations were similar between respondents in the two groups (ranging between 1 to 10 on the NRS). The exception was backache (mean 7.17 vs 5.66, p = 0.015) and joint pain (mean 7.2 vs 5.98, p = 0.018), which were higher in the chronic NP group (Table 4).

**Table 3:** Pain characteristics of pain respondents.

	Neuropathic	Non-neuropathic	
No. of pain sites with NRS score ≥ 7	Responses in %		P value
0	23.8	49.5	0.0001
1	41.3	33	0.1579
2	33.8	16.2	0.0009
**Average pain category**			
(mild, NRS 1 to 3)	2	9	0.0412
(moderate, NRS 4 to 6)	22	41	0.0117
(severe, NRS 7 to 10)	76	50	0.0041
**Highest/Average pain score (NRS)**	**Mean (SD) [range]**		
Highest pain score	7.75 (1.698) [3–10]	6.43 (2.141) [1–10]	0.0001
Average pain score	2.68 (0.612)	2.44 (0.78)	p>0.05
**Pain duration**			
Duration of pain (in months)	58.0 [3–600]	50.3 [0.25–768]	P = 0.035

Values in % and mean (SD)[range].

NRS = Numerical rating scale

**Table 4:** Pain locations and severity of pain.

	Responses in %			Average Pain score (Mean (SD))			Reported as being one of two most painful sites (%)		
Reported pain site	Neuropathic	Non-neuropathic	P value	Neuropathic	Non-neuropathic	P value	Neuropathic	Non-neuropathic	P value
Headache	65	50.8	0.0264	7.44 (1.90)	5.63 (2.37)	0.391	16.3	13.7	0.5963
Backache	66.3	51.5	0.019	7.17 (1.93)	5.66 (1.91)	0.015	27.5	21.9	0.3062
Muscle pain	85	60.8	0.0001	6.74 (2.06)	5.60 (2.28)	0.195	36.3	21.9	0.0096
Joint pain	85	71.9	0.0166	7.20 (2.20)	5.98 (2.25)	0.018	48.8	36.3	0.0435
Toothache	41.3	25.5	0.0061	6.83 (1.33)	5.37 (2.39)	0.321	3.8	5.4	0.7809
Oral/facial pain	27.5	15.5	0.0147	6.25 (3.00)	5.22 (2.64)	0.843	3.8	2.1	0.41
Menstrual pain	11.3	13.1	0.7172	6.00 (1.00)	6.73 (1.87)	0.856	3.8	3.9	1
Stomach ache	50	29.6	0.0006	6.40 (1.52)	4.98 (1.73)	0.268	6.3	9	0.515
Abdominal	42.5	32.5	0.0932	6	4.70 (2.85)	0.504	1.3	5.2	0.229
Others	6.3	7.5	0.8168	7.00 (3.16)	7.25 (2.00)	0.644	2.5	4.1	0.7504

Values in % and mean (SD).

Muscle (36.3% vs 21.9%, p = 0.0096) and joint pain (48.8% vs 36.3%, p = 0.0435) were more frequently reported to be the two most painful sites in respondents with chronic NP (Table 4). Respondents with chronic NP suffered from pain for a longer duration of time compared to those with chronic non-NP ((58.0 [3–60] versus 50.3 [0.25–768] months (p = 0.035)) (Table 3).

### Help sought and satisfaction with treatment

Respondents sought help and received treatment from a number of different professionals. A significantly higher proportion of those with chronic NP consulted a pain specialist (16.3% vs 4.6%, p = 0.0006), a traditional Chinese medical practitioner (40.0% vs 2.63%, p = 0.0203), and a psychiatrist (8.8% vs 2.8%, p = 0.0212) (Table 5). Compared with respondents with chronic NP, significantly more chronic non-NP respondents were prescribed oral medication by healthcare professionals (47.2% vs 60.1%, p = 0.0226), whilst more chronic NP respondents were prescribed cupping (2.4% vs 0.3%, p = 0.0170) (Table 5). Regarding satisfaction with treatment prescribed by health care professionals, significantly fewer respondents with chronic NP felt their treatment was ‘very successful’ (2.9% vs 11.8%, p = 0.0263) (Table 5).

**Table 5:** Professionals consulted, medication received from professionals and satisfaction with prescribed treatment.

Professional consulted	Responses in % (Chronic pain with neuropathic characteristics)	Responses in % (Chronic non-neuropathic pain)	P value
General practitioner	57.5	48.5	0.1765
Specialist	47.5	39.4	0.2116
Chinese medical doctor	40	2.63	0.0203
Physiotherapist	30	22.4	0.151
Bone setter	23.8	15.2	0.0703
Pain specialist	16.3	4.6	0.0006
Psychiatrist	8.8	2.8	0.0212
Not consulted professional	8.8	17	0.0646
Pharmacist	3.8	2.6	0.473
Massage therapist	0	1	-
Others	0	0.5	-
**Treatment/therapy prescribed**			
Oral medication	47.2	60.1	0.0226
Physiotherapy	22.4	25.8	0.5814
Acupuncture	18.4	19.6	0.8244
Surgery	3.2	5.7	1
Topical medication	2.4	7	0.4501
Cupping	2.4	0.3	0.017
Injected medication	1.6	1.3	0.3423
Body check	1.6	3.4	1
Psychotherapy	0.8	1.3	1
Massage	0.8	4.4	0.3338
Dental treatment	0	1.5	-
Bone setter	0	0.5	-
**Level of satisfaction with treatment**			
Very successful	2.9	11.8	0.0263
Helped somewhat	64.3	52.1	0.0833
Didn’t help much	21.4	22.3	1
Didn’t help at all	10	12.8	0.6863
Medical professional did not suggest any treatment	1.4	1	0.564

Values in %

Oral analgesics and rest were the most commonly self-prescribed treatment modalities for both groups (Table 6). Respondents with chronic NP were significantly more likely to obtain oral analgesic medications (67.5% vs 51.0%, p = 0.0094) and nutritional therapy (43.8% vs 29.6%, p = 0.0176). Significantly fewer chronic NP respondents chose ‘no self-help modalities’ (3.8% vs 11.6%, p = 0.0410) (Table 6). There was no difference in the level of satisfaction with self-prescribed treatment modalities between the two groups (Table 6). Neither were there any differences between the two groups in the type of pain medications used (Table 7). Significantly fewer chronic NP respondents found oral analgesic medications ‘very useful’ (3.75% vs 11.8%, p-value 0.0215) (Table 7).

**Table 6:** Respondent self-prescribed treatment and satisfaction with self-prescribed treatment.

Medication type	Responses in % (Chronic pain with neuropathic characteristics)	Responses in % (Chronic non-neuropathic pain)	P value
Oral analgesic	67.5	51	0.0094
Rest	65	58.5	0.3176
Nutritional therapy	43.8	29.6	0.0176
Relaxation techniques	40	34.3	0.3686
Vitamins	20	19.3	0.8774
Topical medication	6.3	5.7	0.7943
No self-help modalities	3.8	11.6	0.041
Massage	2.5	1.8	0.655
Exercise	2.5	2.8	1
Nutritional intake	2.5	1.8	0.655
Others	2.5	3.4	1
Smoking cessation	1.3	2.8	0.7004
Antacids	0	1.5	-
**Level of satisfaction**			
Very successful	8.1	13.9	0.2488
Helped somewhat	74.3	64.2	0.1051
Didn’t help much	13.5	14.2	1
Didn’t help at all	4.1	7.7	0.3263

Values in %

**Table 7:** Oral analgesic medications taken and satisfaction with oral analgesic medication.

	Responses in % (Chronic pain with neuropathic characteristics)	Responses in % (Chronic non-neuropathic pain)	P value
Paracetamol	42.5	39.7	0.7073
Aspirin	10	6.7	0.3416
Anti-rheumatic analgesic	8.8	6.4	0.4658
Anti-depressant	6.3	2.6	0.1518
Addictive analgesics (opioids)	3.8	1	0.1004
NSAID	1.3	2.4	1
Chinese medicine	0	1.3	-
None	41.3	40.2	0.9007
Others	12.6	16	0.72
**Level of satisfaction**			
Very useful	3.75	13.14	0.0215
Somewhat useful	42.5	36.86	0.4552
Not very useful	7.5	4.38	0.2314
Not useful at all	1.25	4.12	0.2199
Did not answer	45	41.49	0.6595

Values in %

### Respondent perceived cause of pain and effect on daily life

The most commonly perceived cause of pain for both chronic non-NP and chronic NP was ‘too much physical activity’ (Table 8). Significantly more respondents with chronic non-NP perceived ‘old age’ as being the cause of pain (1.3% vs 8.2%, p = 0.0278) (Table 8). Respondents with chronic NP were significantly more likely to perceive ‘too much pressure outside of work’ to be the cause of their pain (3.8% vs 0.5%, p = 0.0372) (Table 8). With respect to the effect of pain on work, significantly more chronic NP respondents reported ‘not being able to work again’ (7.4% vs 1.8%, p = 0.0131), and ‘not working’ (50.0% vs 36.3%, p = 0.0239) compared to chronic non-NP respondents (Table 9). Chronic NP respondents were also significantly less likely to report ‘not affect work’ (15.0% vs 36.1%, p = 0.0002) (Table 9). In respondents who were working, there was no significant difference between the numbers of days of sick leave taken between the two groups (Table 9). A significantly higher proportion of respondents with chronic NP reported that their pain ‘affected their daily lives a lot’ (50.0% vs 26.7%, p <0.0001) (Table 9).

**Table 8:** Self-perceived cause of pain.

	Responses in % (Chronic pain with neuropathic characteristics)	Responses in % (Chronic non-neuropathic pain)	P value
Too much physical activity	18.8	18.6	1
Sickness	11.3	7.2	0.2531
Work related injury	10	6.7	0.3416
Posture problems	10	8.2	0.6598
Weak disposition	7.5	7.7	1
Too much pressure at work	5	4.1	0.7605
Too much pressure outside of work	3.8	0.5	0.0372
Dietary problems	2.5	4.1	0.7504
Lack of good quality sleep	2.5	1.3	0.3423
Non-work related injury	1.3	2.1	1
Environmental problems	1.3	1.8	1
Mental pressure	1.3	0.5	0.4309
Old age	1.3	8.2	0.0278
Overweight	0	1.3	-
Pregnancy	0	0.5	-
Smoking	0	0.5	-
Allergies	0	1	-
Menstrual problems	0	1	-
Fatigue	0	0.3	-
Weather	0	0.8	-
Old injury	0	1.3	-
Lack of exercise	0	1	-
Working long hours	0	0.3	-
Exercise	0	0.8	-
Dental cavities	0	1	-
Wisdom tooth	0	0.3	-
Tooth Structure	0	0.3	-
Medication induced	0	0.3	-
Hunger	0	0.3	-
Miscellaneous reasons	3.8	1.8	0.3857
Others (not stated)	12.1	6.4	0.2
Don’t know	7.5	9.5	0.6745
Did not reply	0	0.3	-

Values in %

**Table 9:** Effect of pain on work and daily life.

Effect of pain on work	Responses in % (Chronic pain with neuropathic characteristics)	Responses in % (Chronic non-neuropathic pain)	P value
Not working	50	36.3	0.0239
Not affecting work	15	36.1	0.0002
Sick leave	13.7	8.8	0.2087
Could not work again	7.4	1.8	0.0131
Change in job nature	3.7	6.7	0.4468
Others	3.7	1	0.1004
Could not concentrate on work	1.3	1.5	1
Lower work efficiency	1.3	2.1	1
Affect mood at work	1.3	0.5	0.4309
Feeling tired at work	1.3	0.5	0.4309
Withstand pain	1.3	0.8	0.5288
Change of job	0	0.5	-
Affect ability to function	0	2.1	-
Resting at work	0	1	-
Did not answer	0	0.3	-
**Number of days taken off work in past year**			
0	81	81.2	1
1 to 10	12.7	15.6	0.838
More than 10	6.3	3.2	0.747
**Effect of pain on daily life**	** **	** **	** **
Affected a lot	50	26.7	0.0001
Affected somewhat	27.5	29.5	0.788
Affected a little	21.3	28.8	0.2152
Not affected at all	1.3	15	0.0002

Values in %

## Discussion

This is the first population-based study comparing outcomes, impact and health seeking behaviour between individuals experiencing chronic pain with neuropathic characteristics (NP) and chronic non-neuropathic pain (non-NP) in a Chinese population. While several population surveys have been done in Western countries, there is a paucity of data evaluating chronic neuropathic pain in Asian populations. In addition to our report, there is one other recent Asian based population survey evaluating the prevalence and impact of chronic neuropathic pain in a Japanese population [15].

In this study, the highest pain score was significantly greater in chronic NP. Other population studies have also reported higher pain severity amongst chronic pain patients with neuropathic characteristics [3, 4, 8, 9, 15]. Since the intensity of chronic pain usually fluctuates, we evaluated both the highest and average pain scores. There was no significant difference in average pain scores between the two groups. However, the mean average NRS pain scores for both groups was below 3/10, which corresponds to a low intensity of pain [16], and is unlikely to significantly affect quality of life. Instead, the highest pain scores, which corresponded to moderate to severe pain, is more likely to significantly impact individuals. Duration of pain was significantly longer and number of pain sites with an NRS score over 7/10 was higher in respondents with chronic NP. These finding are also similar to most other population studies [3, 4, 7, 8]. The results here show that chronic NP was associated with worse outcomes in terms of pain intensity and duration amongst Chinese patients in Hong Kong. The reason for this is unclear. It may be due to a difference in underlying pathological process of neuropathic pain compared to non-neuropathic pain. Another explanation is that treatment for chronic neuropathic pain may be less effective, thus resulting in worse outcomes. Apart from pain severity and duration, chronic neuropathic pain has been shown by others to have a greater negative impact on function, quality of life, sleep, and mental health compared with chronic non-neuropathic pain [3, 9, 15]. Similar to these studies, we found that chronic NP was associated with greater negative impact on work and daily life. This may be due to the greater pain severity associated with chronic NP. In addition, the quality of neuropathic pain itself may also lead to increased impairment. The characteristic of neuropathic pain has been shown to negatively impair physical and mental quality of life independent of pain intensity and duration [3].

A significantly higher proportion of respondents with chronic NP consulted a pain specialist, traditional Chinese medical practitioner, and psychiatrist. Chronic neuropathic pain is difficult to treat, and is associated with more severe and prolonged pain. Therefore individuals with chronic neuropathic pain are probably more likely to be referred to a pain specialist. The percentage of respondents with chronic NP and non-NP who consulted a pain specialist was 16.3% and 4.6%, respectively. This shows that the majority of patients with chronic pain regardless of the type of pain did not receive input from a pain specialist. This may be partly because many of the respondents only had pain of mild intensity, which did not warrant pain specialist consultation. However, 50% of the respondents with chronic NP in this survey reported that their daily life was affected a lot by their pain. Therefore, this low figure more likely reflects a lack of awareness of the availability of pain specialist amongst the general public and other health care professionals. Pain specialists are able to implement a more holistic, bio-psychosocial approach, which is particularly important for the management of patients with complex pain problems. Patients with chronic pain, especially those with chronic neuropathic pain, may benefit more with additional pain specialist input. It is important to increase the awareness of other healthcare professionals about the availability and work of pain specialists in order to improve the quality of management for these patients. We also found that a significantly greater proportion of respondents with chronic NP sought help from a traditional Chinese medical practitioner. Traditional Chinese medicine is a popular option amongst Chinese people, and is also becoming more popular in Western countries. Chinese medical formulations such as aconitum may improve neuropathic pain, but overall there is little evidence to support the use of traditional Chinese medicine for neuropathic pain [17]. A recent Cochrane review concluded that there is currently insufficient evidence to support or refute the use of acupuncture for neuropathic pain [18]. Despite the lack of supporting evidence, the popularity of traditional Chinese medicine for chronic NP observed here may be due to cultural influences and the difficulty in effectively reducing neuropathic pain with conventional treatment [1, 19]. Respondents with chronic NP were also more likely to have seen a psychiatrist, suggesting a greater association with psychiatric issues. This appears to agree with results from a French general population survey, where respondents experiencing chronic pain with neuropathic characteristics had worse depression and anxiety scores compared to those without neuropathic characteristics [3]. Pain and depression have a bidirectional relationship, and a higher pain severity is linked with more severe depression [20]. It is possible that higher pain intensity contributed to more severe depression in individuals with chronic NP, resulting in more psychiatric consultations. However, this explanation may be over simplistic, and shared biological mechanisms between neuropathic pain and depression may be present.

Respondents with chronic NP were significantly less likely to be prescribed oral analgesics drugs by health care professionals. This is in contrast to results from other surveys, which showed a higher analgesic prescription rate for chronic NP [3, 15]. In addition, there was no difference in the type of oral analgesic drugs taken between the two groups. Pharmacological treatment of neuropathic pain is different from non-neuropathic pain [1, 19]. However, the most common oral analgesic taken by both group of respondents in this survey was paracetamol. These results suggest that chronic NP in Hong Kong was undertreated, and many patients did not receive the most appropriate analgesics. Pharmacological treatment with oral medication with anti-neuropathic pain medication remains an integral part of neuropathic pain management [1]. Adequate training of health care professionals is needed to increase the prescription of appropriate anti-neuropathic medication for chronic neuropathic pain.

We found that female sex, working part time, being a full-time student, and feeling ‘very much under pressure’ were associated with a significantly higher risk of chronic NP. Female gender and feeling under pressure were also found to be risk factors for chronic pain in our previous report in a Chinese population, and are therefore not specific to chronic NP [10]. Interestingly, being a full-time student and working part time was not a risk factor for having chronic pain in our previous report, suggesting that these may be specific to chronic NP [10].

‘Too much physical activity’ was the most commonly perceived cause of pain for both types of pain, and there were no differences between the two groups. Respondents with chronic NP were significantly more likely to perceive ‘too much pressure outside of work’ as a cause for pain. On the other hand, there were no differences between the two groups with regards to ‘too much pressure at work’. Although more respondents with chronic NP thought ‘too much pressure outside of work’ were a cause for pain, the actual figure was only 3.8%. This may be an incidental finding. In an animal study, chronic stress was shown to worsen neuropathic pain [21]. However, if mental pressure was associated specifically with neuropathic pain, then a significant difference should also be observed with ‘too much pressure at work’.

One of the limitations of this study is the low response rate (32.32%). This is lower than the response rate of some other previous population surveys for neuropathic pain, which had response rates of over 50% [9, 15]. This lower response rate should not have affected the power of the study, but may have introduced selection bias. Another limitation is that telephone calls were made in evening hours, and therefore individuals who work at night are likely to be under-represented. People who no longer use their home phones are also likely to be under-represented since only landline phone numbers were used. Similar to other population surveys on neuropathic pain, we have used a validated neuropathic pain questionnaire to identify individuals suffering from pain with neuropathic characteristics. However, a definite diagnosis of neuropathic pain requires a combination of positive history, physical signs, and confirmatory tests, which is not possible in a population survey [1]. Furthermore, while telephone surveys on neuropathic pain have been conducted in the past [22, 23], assessment of neuropathic pain can be difficult via telephone call. Therefore, it is possible that some individuals with true chronic neuropathic pain were missed. Finally, a telephone survey in a large metropolis in Hong Kong may not be fully representative of the whole of China. However, Hong Kong should be similar to other major cities in this rapidly developing country.

In conclusion, we report that individuals with chronic NP have greater pain severity, longer pain duration, and greater negative impact on work and daily activities compared with individuals with chronic non-NP in a Chinese population. This is in agreement with results from surveys in other populations. Individuals with chronic NP were less likely to be satisfied with prescribed treatment and less likely to find oral analgesics effective. They were also less likely to be prescribed oral analgesics by healthcare professionals, and there was no distinction between the two groups in terms of type of oral analgesic taken. Chronic NP is a significant health care burden amongst Chinese individuals in Hong Kong and appears to be inadequately managed. Education for health care professionals and the general public is necessary to correctly identify and treat chronic neuropathic pain as a distinct entity.

## Supporting information

S1 Appendix Full questionnaire survey in English. (DOCX)

S2 Appendix Full questionnaire survey in Chinese. (DOCX)
