# Supplementary material for: A comparison of chronic pain with and without neuropathic characteristics in a Hong Kong Chinese population: An analysis of pain related outcomes and patient help seeking behaviour
Source: PLoS One. 2018 Oct 24;13(10):e0204054. doi: 10.1371/journal.pone.0204054 (PMC6200186; doi:10.1371/journal.pone.0204054)
Supplement: S1 Appendix — (DOCX) [file pone.0204054.s001.docx]

**Supporting Information Appendix 1: Full questionnaire survey in English**

*Part A.*

Q1. Gender:

Male = 1

Female = 2

Q2a. Have you had any of the following pains in the past 12 months?

1. Headache

2. Back Pain

3. Muscle Pain

4. Joint Pain

5. Toothache

6. Orofacial Pain

7. Menstrual Pain

8. Stomachache

9. Intestinal Pain

10. Others ________________

No – go to Part B

Yes – mark down on Q5 table

Q2b. Of the pains you mentioned just now, which two would you consider the most severe?

Q3a. Have the two pains you just mentioned ever persisted for three months or more?

Yes =1

No = 2

Q3b. How long ago did these two pains start? *Answers coded in months*

*Pain 1:________________*

*Pain 2:________________*

Q3c. On how many days have these two pains been present in the past 12 months? (mark down on Q5 table)

1 to 5 =1

6 to 10 =2

11 to 30 =3

31 to 100 =4

101 or more days=5

None = 6

Unknown = 7

Q4a. Did the pain feel like pins and needles?

Yes = 1 No = 0

Q4b. Did the pain feel hot/burning?

Yes = 1 No = 0

Q4c. Did the pain feel numb?

Yes = 1 No = 0

Q4d. Did the pain feel like electric shocks?

Yes = 1 No = 0

Q4e. Was the pain made worse with the touch of clothing or bedsheets?

Yes = 1 No = 0

Q4f. Was the pain limited to your joints?

Yes = 1 No = 0

Q5. Please give a score from 1 to 10 to represent the intensity of your pain, with 1 being slight pain, and 10 being the worst pain imaginable.

|  | *Headache* | *Muscle Pain* | *Back Pain* | *Joint Pain* | *Tooth-ache* | *Orofacial Pain* | *Menstrual Pain* | *Stomach-ache* | *Intestinal Pain* | Others |
| --- | --- | --- | --- | --- | --- | --- | --- | --- | --- | --- |
| *Q3c* |  |  |  |  |  |  |  |  |  |  |
| *Q5* |  |  |  |  |  |  |  |  |  |  |

Q6. What do you think is the main cause of your pain? (mark down on Q10 table)

Too much physical activity =1

pressure from work = 2

pressure not related to work = 3

work-related injury = 4

not work-related injury = 5

sickness = 6

weak body = 7

over-weight = 8

pregnancy = 9

posture = 10

dietary problems = 11

smoking = 12

alcohol = 13

environmental concerns (home, office) = 14

allergies = 15

unknown = 16

Q7. Has your employment been affected by your pain in the past 12 months? (mark down on Q10 table)

unemployed = 1

work has not been affected at all = 2

had to adjust work = 3

took sick leave = 4

changed jobs = 5

was not able to continue working = 6

Q8. In the past 12 months, how many days of sick leave have you had to take due to your pain? (mark down on Q10 table)

Q9. Please give a score from 1 to 4 to represent how seriously your pain has affected your daily life, with 1 being severely affected, and 4 being not affected at all. (mark down on Q10 table)

Severely affected = 1

Moderately affected = 2

A little bit affected = 3

Not affected at all = 4

Q10. Have you filed any litigation because of this pain?

No = 1

Yes, in process = 2

Yes, completed = 3

| Pain | *Headache* | *Muscle Pain* | *Back Pain* | *Joint Pain* | *Tooth-ache* | *Orofacial Pain* | *Menstrual Pain* | *Stomach-ache* | *Intestinal Pain* | Others |
| --- | --- | --- | --- | --- | --- | --- | --- | --- | --- | --- |
| *Q6* |  |  |  |  |  |  |  |  |  |  |
| *Q7* |  |  |  |  |  |  |  |  |  |  |
| *Q8* |  |  |  |  |  |  |  |  |  |  |
| *Q9* |  |  |  |  |  |  |  |  |  |  |
| *Q10* |  |  |  |  |  |  |  |  |  |  |

Q11. In the past 12 months, have you visited any of the following because of this pain?

Yes = 1 (go to Q11a)

No = 2 (go to Q12)

| Pain | *Headache* | *Muscle Pain* | *Back Pain* | *Joint Pain* | *Tooth-ache* | *Orofacial Pain* | *Menstrual Pain* | *Stomach-ache* | *Intestinal Pain* | Others |
| --- | --- | --- | --- | --- | --- | --- | --- | --- | --- | --- |
| *general practitioner* |  |  |  |  |  |  |  |  |  |  |
| *specialist doctor* |  |  |  |  |  |  |  |  |  |  |
| *pain specialist* |  |  |  |  |  |  |  |  |  |  |
| *pharmacist* |  |  |  |  |  |  |  |  |  |  |
| *physiotherapist* |  |  |  |  |  |  |  |  |  |  |
| *traditional Chinese medical practitioner* |  |  |  |  |  |  |  |  |  |  |
| *bonesetter* |  |  |  |  |  |  |  |  |  |  |
| *psychiatrist* |  |  |  |  |  |  |  |  |  |  |
| *psychologist* |  |  |  |  |  |  |  |  |  |  |
| *others* |  |  |  |  |  |  |  |  |  |  |

Q11a. What treatment have you received from these people?

| Pain | *Headache* | *Muscle Pain* | *Back Pain* | *Joint Pain* | *Tooth-ache* | *Orofacial Pain* | *Menstrual Pain* | *Stomach-ache* | *Intestinal Pain* | Others |
| --- | --- | --- | --- | --- | --- | --- | --- | --- | --- | --- |
| *oral medication* |  |  |  |  |  |  |  |  |  |  |
| *surgical procedures* |  |  |  |  |  |  |  |  |  |  |
| *physiotherapy* |  |  |  |  |  |  |  |  |  |  |
| *physiotherapy* |  |  |  |  |  |  |  |  |  |  |
| *acupuncture* |  |  |  |  |  |  |  |  |  |  |
| *counselling* |  |  |  |  |  |  |  |  |  |  |
| *others* |  |  |  |  |  |  |  |  |  |  |

Q11b. How would you rate the effectiveness of the treatment you have been offered (mark down on Q11a table)?

very successful = 1

helped somewhat = 2

not much effectiveness = 3

not at all effective = 4

was recommended no treatment =5

Q12. In the past 12 months, have you undergone any treatment yourself because of your pain problem? How would you rate the effectiveness of the treatment you have undergone yourself?

Not done = 0

very successful = 1

helped somewhat = 2

not much effectiveness = 3

not at all effective = 4

| Pain | *Headache* | *Muscle Pain* | *Back Pain* | *Joint Pain* | *Tooth-ache* | *Orofacial Pain* | *Menstrual Pain* | *Stomach-ache* | *Intestinal Pain* | Others |
| --- | --- | --- | --- | --- | --- | --- | --- | --- | --- | --- |
| *oral analgesics* |  |  |  |  |  |  |  |  |  |  |
| *rest* |  |  |  |  |  |  |  |  |  |  |
| *learn relaxation techniques* |  |  |  |  |  |  |  |  |  |  |
| *dietary changes* |  |  |  |  |  |  |  |  |  |  |
| *smoking cessation* |  |  |  |  |  |  |  |  |  |  |
| *take vitamins* |  |  |  |  |  |  |  |  |  |  |
| *others* |  |  |  |  |  |  |  |  |  |  |

Q13. Have you ever used any of the following prescribed analgesics? How effective did you find the analgesics?

Not used = 0

very successful = 1

helped somewhat = 2

not much effectiveness = 3

not at all effective = 4

| Pain | *Headache* | *Muscle Pain* | *Back Pain* | *Joint Pain* | *Tooth-ache* | *Orofacial Pain* | *Menstrual Pain* | *Stomach-ache* | *Intestinal Pain* | Others |
| --- | --- | --- | --- | --- | --- | --- | --- | --- | --- | --- |
| *Panadol* |  |  |  |  |  |  |  |  |  |  |
| *aspirin* |  |  |  |  |  |  |  |  |  |  |
| *NSAID* |  |  |  |  |  |  |  |  |  |  |
| *opioids* |  |  |  |  |  |  |  |  |  |  |
| *anti-depressants* |  |  |  |  |  |  |  |  |  |  |
| *others* |  |  |  |  |  |  |  |  |  |  |

**Part B.** Demographics

Q14. Age _______________

Q15. Marital status

Single =1

Married/ Cohabitation = 2

Divorced = 3

Widowed = 4

Q16. Education level

Uneducated = 1

Primary school = 2

Secondary school (not completed) = 3

Secondary school (graduated) = 4

Matriculation = 5

Tertiary = 6

Q17. Job type

Full time = 1

Part time = 2

Unemployment = 3

Housewife = 4

Student = 5

Student (Part time) = 6

Retired = 7

Unemployed due to sick = 8

Q18. Current Occupation ___________________________

Q19. Compare to general, how would you rate your current living pressure?

much larger than general = 1

larger than general = 2

similar to general = 3

less than general = 4

**END OF QUESTIONNAIRE**

**Thank you for your time and Good Bye**
