# Supplementary material for: A comparison of chronic pain with and without neuropathic characteristics in a Hong Kong Chinese population: An analysis of pain related outcomes and patient help seeking behaviour
Source: PLoS One. 2018 Oct 24;13(10):e0204054. doi: 10.1371/journal.pone.0204054 (PMC6200186; doi:10.1371/journal.pone.0204054)
Supplement: S2 Appendix — (DOCX) [file pone.0204054.s002.docx]

**Supporting Information Appendix 2: Full questionnaire survey in Chinese**

Part A.

Q1. 男性=1 女性=2

Q2a. 由現在計起，過去的12個月內，您有否下面的痛呢？

1. 頭痛

2. 背痛

3. 肌肉痛

4. 關節痛

5. 牙痛

6. 口腔面頰

7. 月經痛(只適用於女性)

8. 胃痛

9. 肚痛

10. 其他痛症 _________________

是 🡪 在Q5的列表記錄痛症的種類

否 🡪 前往Part B

Q2b. (如痛症的種類超過兩種)在上述的痛症之中，有哪兩種是比較嚴重？

Q3a. 這些痛是否有維持超過三個月或以上?

是 = 1

否 = 2

Q3b. 這些痛已存在多久?

第一種痛 _________________

第二種痛 _________________

Q3c. 在過去的12個月，這些痛大概會痛幾多日呢？

(將兩種較嚴重痛症維持的日數記錄在Q5的列表)

1 – 5 =1

6 – 10 =2

11 – 30 =3

31 – 100 =4

101或以上 =5

沒有 =6

不確定 =7

Q4a. 您的痛楚是否好像被針剌般疼痛？

是=1 否=0

Q4b. 您的痛楚是否灼熱或好像被火燒一樣？

是=1 否=0

Q4c. 您的痛楚是否帶有痲痺感？

是=1 否=0

Q4d. 您的痛楚是否好像觸電一樣？

是=1 否=0

Q4e. 您的痛楚是否因觸碰衣服或床單而加劇？

是=1 否=0

Q4f. 您的痛楚是否只限於關節部位？

是=-1 否=0

Q5. 如果用1至10來代表痛的嚴重程度，1代表最小的痛，而10代表最大的痛，您認為這些痛有幾分呢？ (在以下列表記錄痛的程度)

|  | 頭痛 | 背痛 | 肌肉痛 | 關節痛 | 牙痛 | 口腔面頰 | 月經痛 | 胃痛 | 肚痛 | 其他 |
| --- | --- | --- | --- | --- | --- | --- | --- | --- | --- | --- |
| 維持日數 |  |  |  |  |  |  |  |  |  |  |
| 嚴重程度 |  |  |  |  |  |  |  |  |  |  |

Q6. 您認為導致您的痛最主要原因是甚麼呢？

體力勞動 =1

上班時的精神壓力 =2

非上班時的精神壓力 =3

工作時受傷 =4

非工作時受傷 =5

有病 =6

身體弱 =7

過重 =8

懷孕 =9

姿勢有問題 =10

飲食有問題 =11

吸煙 =12

飲酒 =13

環境欠佳(居住、工作) =14

敏感 =15

不知道 =16

Q7. 在過去的12個月，您這些痛有沒有影響您的工作呢？

(在Q10的列表記錄工作能力)

無工作 =1

工作不受影響 =2

要改變工作範圍 =3

要放病假 =4

要轉工 =5

以後不能再工作 =6

Q8. 在過去的12個月，您有幾多日因為以上的痛而請病假呢？

(在Q10 的列表記錄病假的日數)

Q9. 您認為這些痛有否影響您的日常生活，例如吃飯、去街、看電視、戶外活動等等？

(在Q10的列表記錄對日常生活的影響)

大受影響 =1

有影響 =2

些少影響 =3

完全無影響 =4

Q10. 您有否因為您以上提過的痛而提出法律訴訟呢？

否 =1

是，正在進行中 =2

是，已完成訴訟 =3

|  | 頭痛 | 背痛 | 肌肉痛 | 關節痛 | 牙痛 | 口腔面頰 | 月經痛 | 胃痛 | 肚痛 | 其他 |
| --- | --- | --- | --- | --- | --- | --- | --- | --- | --- | --- |
| 成因 |  |  |  |  |  |  |  |  |  |  |
| 影響工作 |  |  |  |  |  |  |  |  |  |  |
| 要請病假 |  |  |  |  |  |  |  |  |  |  |
| 影響日常生活 |  |  |  |  |  |  |  |  |  |  |
| 追究法律責任 |  |  |  |  |  |  |  |  |  |  |

Q11. 在過去的12個月，您有否因為以上提過的痛而去看過下列人士？

是 =1

否 =2

(如果是，前往Q11a)

(如果否，前往Q12)

|  | 頭痛 | 背痛 | 肌肉痛 | 關節痛 | 牙痛 | 口腔面頰 | 月經痛 | 胃痛 | 肚痛 | 其他 |
| --- | --- | --- | --- | --- | --- | --- | --- | --- | --- | --- |
| 普通科醫生 |  |  |  |  |  |  |  |  |  |  |
| 專科醫生 |  |  |  |  |  |  |  |  |  |  |
| 痛症醫生 |  |  |  |  |  |  |  |  |  |  |
| 藥劑師 |  |  |  |  |  |  |  |  |  |  |
| 物理治療 |  |  |  |  |  |  |  |  |  |  |
| 中醫 |  |  |  |  |  |  |  |  |  |  |
| 跌打 |  |  |  |  |  |  |  |  |  |  |
| 精神科醫生 |  |  |  |  |  |  |  |  |  |  |
| 心理學家 |  |  |  |  |  |  |  |  |  |  |
| 其他 |  |  |  |  |  |  |  |  |  |  |

Q11a. 那些人士為您進行了哪一種治療？

|  | 頭痛 | 背痛 | 肌肉痛 | 關節痛 | 牙痛 | 口腔面頰 | 月經痛 | 胃痛 | 肚痛 | 其他 |
| --- | --- | --- | --- | --- | --- | --- | --- | --- | --- | --- |
| 食藥 |  |  |  |  |  |  |  |  |  |  |
| 手術 |  |  |  |  |  |  |  |  |  |  |
| 物理治療 |  |  |  |  |  |  |  |  |  |  |
| 針灸 |  |  |  |  |  |  |  |  |  |  |
| 心理治療 |  |  |  |  |  |  |  |  |  |  |
| 其他 |  |  |  |  |  |  |  |  |  |  |

Q11b. 您認為您接受的治療能否成功地解決痛的問題？

非常成功 =1

有啲幫助 =2

唔係幾成功 =3

完全無效 =4

醫生建議不用治療 =5

Q12. 在過去12個月，您有否因為以上提過的痛而自己進行以下治療，(如有)它們的效果怎樣？

無試 =0

非常成功 =1

有啲幫助 =2

唔係幾成功 =3

完全無效 =4

|  | 頭痛 | 背痛 | 肌肉痛 | 關節痛 | 牙痛 | 口腔面頰 | 月經痛 | 胃痛 | 肚痛 | 其他 |
| --- | --- | --- | --- | --- | --- | --- | --- | --- | --- | --- |
| 食止痛藥 |  |  |  |  |  |  |  |  |  |  |
| 休息 |  |  |  |  |  |  |  |  |  |  |
| 學習放鬆技巧 |  |  |  |  |  |  |  |  |  |  |
| 戒口/食療 |  |  |  |  |  |  |  |  |  |  |
| 戒煙 |  |  |  |  |  |  |  |  |  |  |
| 食維他命 |  |  |  |  |  |  |  |  |  |  |
| 其他 |  |  |  |  |  |  |  |  |  |  |

Q13. 您有否用過止痛藥物？您認為它們的效果如何？

很有效 =1

有啲效 =2

唔係幾有效 =3

完全無效 =4

|  | 頭痛 | 背痛 | 肌肉痛 | 關節痛 | 牙痛 | 口腔面頰 | 月經痛 | 胃痛 | 肚痛 | 其他 |
| --- | --- | --- | --- | --- | --- | --- | --- | --- | --- | --- |
| 必理痛 |  |  |  |  |  |  |  |  |  |  |
| 亞士匹靈 |  |  |  |  |  |  |  |  |  |  |
| 風濕類止痛藥 |  |  |  |  |  |  |  |  |  |  |
| 可上癮的止痛藥 |  |  |  |  |  |  |  |  |  |  |
| 神經痛藥例如抗抑鬱藥或抗癲癇藥 |  |  |  |  |  |  |  |  |  |  |
| 其他 |  |  |  |  |  |  |  |  |  |  |

Part B. **基本資料**

Q14. 您今年幾歲？ _______

Q15. 您的婚姻狀況: _______

獨身 =1

已婚/同居 =2

離婚 =3

鱌寡 =4

Q16. 您的教育程度: _______

未上過學 =1

小學畢業 =2

未完成中學 =3

中學畢業 =4

預料 =5

大專 =6

Q17. 您的工作情況: ______

全職 =1

兼職 =2

失業 =3

家務 =4

學生 =5

學生(非全日制) =6

退休 =7

因身體唔好不能工作 =8

Q18. 請問您做甚麼工作？

_______________________________________________

Q19. 與一般人比較，您認為自己的生活壓力有多大？

比一般人壓力大得多 =1

比一般人壓力大 =2

差不多 =3

比一般人壓力少 =4

**問卷完，多謝您的幫忙，再見**
